# Two-dimensional NMR data of a water-soluble β-(1→3, 1→6)-glucan from *Aureobasidium pullulans* and schizophyllan from *Schizophyllum commune*

**DOI:** 10.1016/j.dib.2017.09.067

**Published:** 2017-10-01

**Authors:** Hiroyuki Kono, Nobuhiro Kondo, Katsuki Hirabayashi, Makoto Ogata, Kazuhide Totani, Shinya Ikematsu, Mitsumasa Osada

**Affiliations:** a Division of Applied Chemistry and Biochemistry, National Institute of Technology, Tomakomai College, Nishikioka 443, Tomakomai, Hokkaido 059 1275, Japan; b Itochu Sugar Co. Ltd., Tamatsuura 3, Hekinan, Aichi 447 8506, Japan; c Department of Chemistry and Biochemistry, National Institute of Technology, Fukushima College, Nagao 30, Iwaki, Fukushima 970 8034, Japan; d Division of Chemical Engineering and Biotechnology, Department of Engineering for Future Innovation, National Institute of Technology, Ichinoseki College, Takanashi, Hagisho, Ichinoseki, Iwate 021 8511, Japan; e Department of Bioresources Engineering, National Institute of Technology, Okinawa College, Henoko 905, Nago, Okinawa 905 2192, Japan; f Department of Chemistry and Materials, Faculty of Textile Science and Technology, Shinshu University, 3-15-1, Tokida, Ueda, Nagano 386 8567, Japan

**Keywords:** NMR, β-(1→3, 1→6)-glucan, *Aureobasidium pullulans*, Schizophyllan, Spectral data

## Abstract

This article contains two-dimensional (2D) NMR experimental data, obtained by the Bruker BioSpin 500 MHz NMR spectrometer (Germany) which can used for the determination of primary structures of schizophyllan from *Schizophyllum commune* (SPG) and a water-soluble β-(1→3, 1→6)-glucan from *Aureobasidium pullulans*. Data include analyzed the 2D NMR spectra of these β-glucans, which are related to the subject of an article in *Carbohydrate Polymers*, entitled “NMR spectroscopic structural characterization of a water-soluble β-(1→3, 1→6)-glucan from *A. pullulans*” (Kono et al., 2017) [1]. Data can help to assign the ^1^H and ^13^C chemical shifts of the structurally complex polysaccharides.

**Specifications Table**

**Table:** 

Subject area	*Chemistry*
More specific subject area	*Structural analysis*
Type of data	*NMR spectra*
How data was acquired	*NMR, Bruker BioSpin AVIII 500 MHz spectrometer*
Data format	*Analyzed*
Experimental factors	*About 30 mg of each sample dissolved in 600 μL of 99.9% dimethylsulfoxide (DMSO)-d*_*6*_.
Experimental features	*All NMR experiments were performed at 363 K.*
Data source location	*National Institute of Technology, Tomakomai College, Nishikioka 443, Tomakomai, Hokkaido 059 1275, Japan*
Data accessibility	*Data are with this article.*

**Value of the data**

• The following data detail NMR characterization of a novel water-soluble β-(1→3, 1→6)-glucan and schizophyllan from *Schizophyllum commune.*
• The NMR data can be helpful to estimate the branching patterns of other β-glucans.
• NMR parameters for the data can be useful for structural characterization of complex polysaccharides.

## 1 Data

**Fig. 1:**
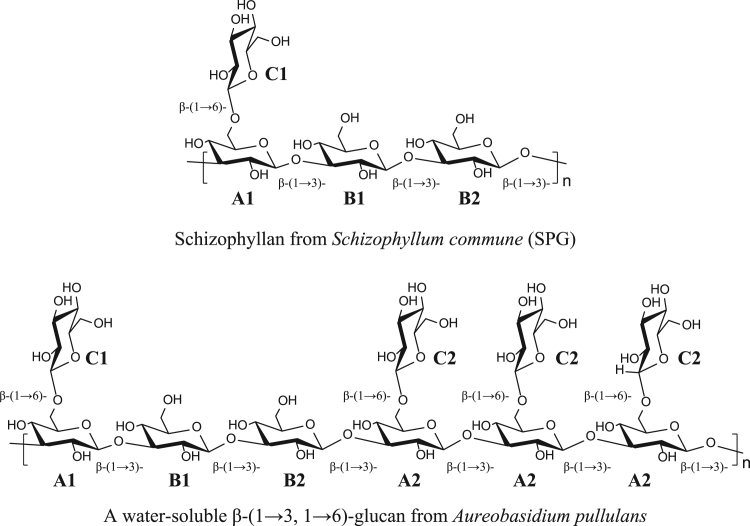
Primary structures of schizophyllan (SPG) and the water-soluble β-(1→3, 1→6)-glucan from *A. pullulans*. The **A1**, **B1**, **B2**, and **C1** residues in SPG and **A1**, **A2**, **B1**, **B2**, **C1**, and **C2** residues in the β-(1→3, 1→6)-glucan are magnetically inequivalent in their structures.

**Fig. 2:**
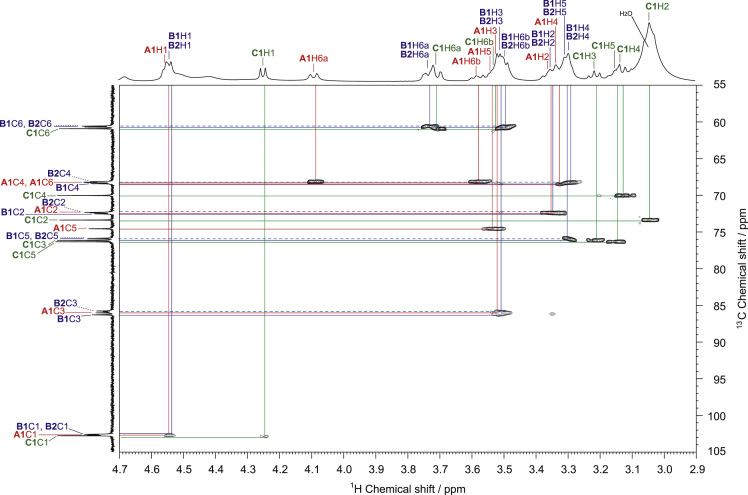
HSQC spectrum of SPG in DMSO-*d*_*6*_ at 363 K. The vicinal ^1^H–^13^C spin couplings of the **A1**, **B1**, **B2**, and **C1** residues in SPG (Fig. 1) are denoted by solid red, solid and dashed blue, and solid green lines, respectively. ^1^H and ^13^C NMR spectra of SPG are shown in horizontal and vertical axes in the HSQC spectrum, respectively, and the ^1^H and ^13^C resonance assignments are indicated in the ^1^H and ^13^C spectra.

**Fig. 3:**
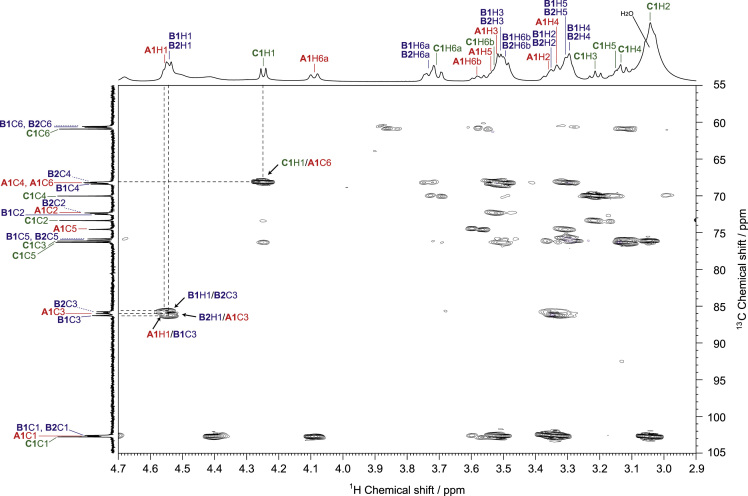
HMBC spectrum of SPG in DMSO-*d*_*6*_ at 363 K. The arrows indicate the interresidual correlations between **A1**H1–**B1**C3, **B1**H1–**B2**C3, **B2**H1–**A3**C3, and **C1**H1–**A1**C6 via glycosidic bonds.

**Fig. 4:**
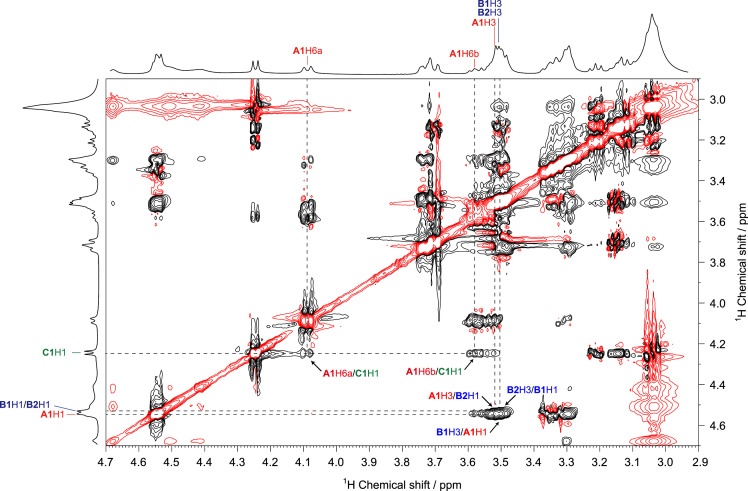
ROESY spectrum of SPG in DMSO-*d*_*6*_ at 363 K. The arrows indicate the interresidual correlations between **B1**H3–**A1**H1, **A1**H3–**B2**H1, **B2**H3–**B1**H1, **A1**H6a–**C1**H1, and **A1**H6b–**C1**H1 via glycosidic bonds.

**Fig. 5:**
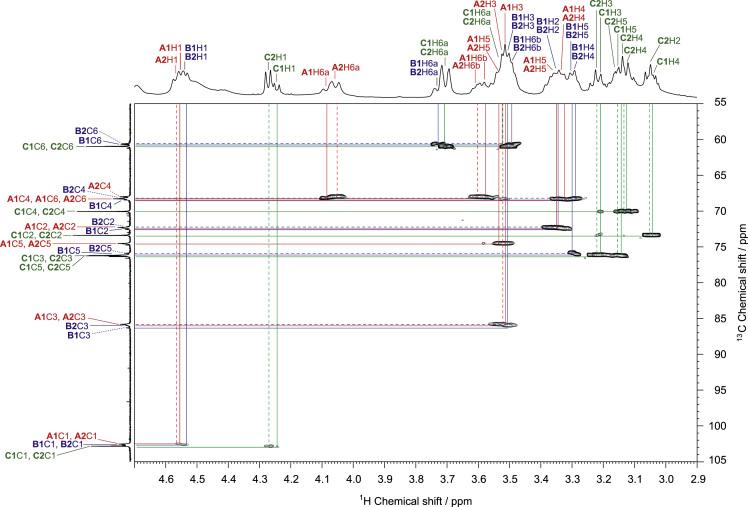
HSQC spectrum of the water-soluble β-(1→3, 1→6)-glucan from *A. pullulans* in DMSO-*d*_*6*_ at 363 K. The vicinal ^1^H–^13^C spin couplings of the **A1**, **A2**, **B1**, **B2**, **C1**, and **C2** residues in the β-(1→3, 1→6)-glucan (Fig. 1) are denoted by solid and dashed red, solid and dashed blue, and solid and dashed green lines, respectively. ^1^H and ^13^C NMR spectra of the β-(1→3, 1→6)-glucan are shown in horizontal and vertical axes in the HSQC spectrum, respectively, and the ^1^H and ^13^C resonance assignments are indicated in the ^1^H and ^13^C spectra.

**Fig. 6:**
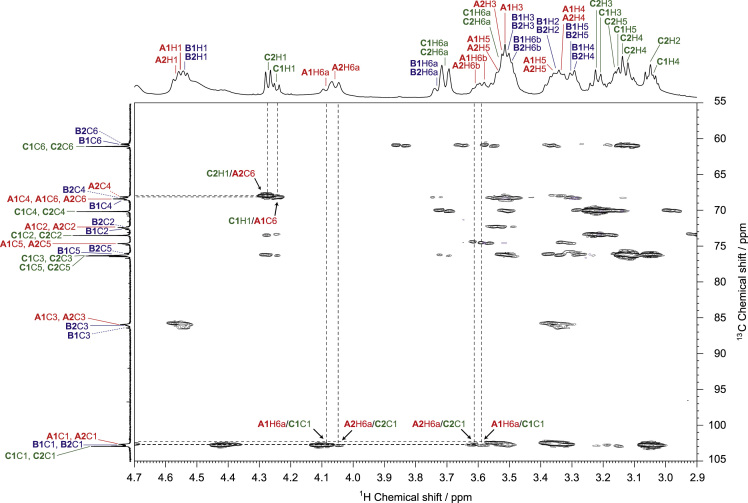
HMBC spectrum of the water-soluble β-(1→3, 1→6)-glucan from *A. pullulans* in DMSO-*d*_*6*_ at 363 K. The arrows indicate the inter-residual correlations between **C1**C1–**A1**H6a, **C1**C1–**A1**H6b, **C2**C1–**A2**H6a, **C2**C1–**A2**H6b, **C1**H1–**A1**C6, and **C2**H1–**A2**C6 via glycosidic bonds.

**Fig. 7:**
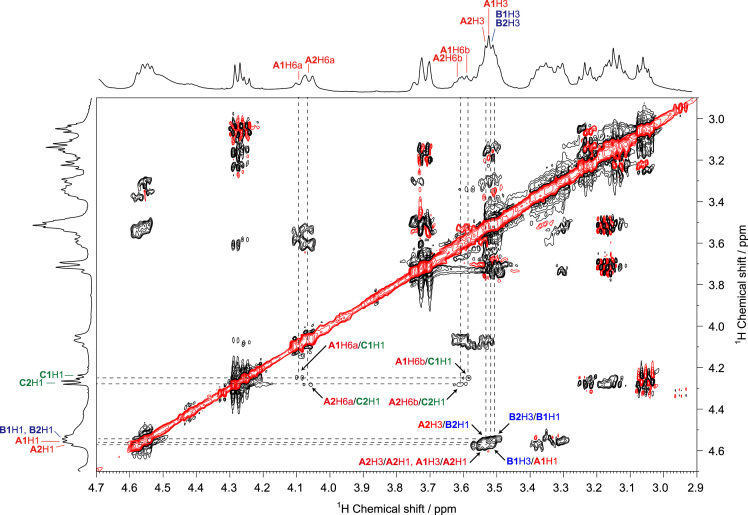
ROESY spectrum of the water-soluble β-(1→3, 1→6)-glucan from *A. pullulans* in DMSO-*d*_*6*_ at 363 K. The arrows indicate the interresidual correlations between **A2**H3–**B2**H1, **A1**H3–**A2**H1, **B1**H3–**A1**H1, **A2**H3–**B2**H1, **B2**H3–**B1**H1, **A2**H6a–**C2**H1, **A2**H6b–**C2**H1, **A1**H6a–**C1**H1, and **A1**H6b–**C1**H1 via glycosidic bonds.

The presented data include 2D NMR spectra of schizophyllan from *Schizophyllum commune* (SPG) and a water-soluble β-(1→3, 1→6)-glucan from *Aureobasidium pullulans* (*A. pullulans*) whose primary structures are shown in Fig. 1. ^1^H–^13^C heteronuclear single quantum coherence (HSQC), 2D ^1^H–^13^C heteronuclear multiple-bond correlation (HMBC), and 2D ^1^H–^1^H rotating frame Overhauser effect spectroscopy (ROESY) spectra of SPG are shown in Fig. 2, Fig. 3, Fig. 4, and those of the water-soluble *A. pullulans* β-(1→3, 1→6)-glucan are in Fig. 5, Fig. 6, Fig. 7, respectively.

## 2 Experimental design, materials and methods

The experiment's planning, design, and data processing correspond to the protocol given in Refs. [1], [2].

### 2.1 Samples

SPG was purchased from InvivoGen (USA). The water-soluble *A. pullulans* β-(1→3, 1→6)-glucan was prepared according to a previously reported method [1], [2].

### 2.2 Description of the NMR experiments

Each sample was dissolved in 600 μL of DMSO-*d*_6_ (99.9% isotropic purity, Sigma-Aldrich (USA)). All NMR spectra were recorded on a Bruker AVIII 500 MHz spectrometer at 363 K. HSQC data were acquired on a 2048 × 256-point matrix for the full spectrum, with 96 scans per increment, and the interpulse delay which corresponded to 1/4 *J*_*CH*_ was set to 3.44 ms. HMBC) data were acquired on a 1024 × 256-point matrix for the full spectrum, with 128 scans per increment, and the delay time for the evolution was set to 62.5 ms. ROESY data were acquired on a 2048 × 256-point matrix for the full spectrum with 64 scans per increment, and the mixing time was 200 ms. The repetition time of each 2D NMR experiment was 2 s, and all 2D NMR data were zero-filled to 2k in both dimensions prior to Fourier transformation. ^1^H and ^13^C chemical shifts were calibrated using the methyl resonances of DMSO at 2.52 ppm for ^1^H and 39.52 ppm for ^13^C.
